# Relationships among perceived learning, challenge and affect in a clinical context

**DOI:** 10.1186/s12909-021-02574-2

**Published:** 2021-03-19

**Authors:** J. R. Rudland, C. Jaye, M. Tweed, T. J. Wilkinson

**Affiliations:** 1 Otago Medical School, University of Otago, Wellington, Education Unit, 23A Mein St, Newtown, Wellington, 6242 New Zealand; 2 Department General Practice and Rural Health, University of Otago, Dunedin School of Medicine, Health Sciences, 55 Hanover Street, Dunedin, 9016 New Zealand; 3 Department of Medicine, University of Otago, Wellington, 23A Mein St, Newtown, Wellington, 6242 New Zealand; 4 Otago Medical School, University of Otago, Christchurch, Education Unit, 2 Riccarton Avenue, Christchurch, 8011 New Zealand

**Keywords:** Learning, Stress, Affect, Medical education

## Abstract

**Background:**

Challenge, sometimes perceived as stress, may be beneficial or detrimental to learning but the circumstances when it may be beneficial are not clear. This study looks at the association of challenge with perceived learning and how this might be influenced by affect, context or the type of learning.

**Method:**

The participants, medical students in their first years of experiential clinical exposure, rated specified learning episodes (LEs) on the perceived learning (low to high), challenge (low to high) and affect (feeling positive to negative). Such learning episodes were self-identified or identified by course organisers. Correlations, using Kendall’s tau-b test, were conducted to explore the associations among learning, challenge and affect. In the second stage the types of LEs were then thematically classified in order to determine those that were positive for learning and challenging and/or associated with positive affect.

**Result:**

There were positive correlations between perceived learning and challenge, and between perceived learning and affect for both types of LEs. The circumstances in which challenge (stress) promoted learning were authentic environments, authentic tasks and simulated clinical activities; most requiring a degree of social interaction.

**Conclusion:**

Challenge and positive affect are beneficial in the perception of discrete learning, but are two separate constructs. Ideally both challenge and affect need to operate alongside authentic supportive clinical activities, that by their nature involve others, to maximise perceived learning.

## Background

Stress was originally defined as a non-specific response of a body to demands made on it [1] by a stressor or a stimulus that triggers the feeling of stress. There has been much written about the negative effects of stress in education. The common perception that stress is negative fails to account for the potential beneficial effects that stress may have on learning [2]. The conflation of stress with distress has hindered examination of positive effects of stress [3, 4].

Not all stress is harmful, and some forms of stress can be positive and motivating forces [5]. Stress can enhance memory [6], particularly when an individual’s autonomic state is activated (a physiological stress response) when there can be an improvement of declarative memory [7]. Declarative memory refers to the conscious or voluntary recollection of previously learned information [8]. An association has been found between self-reported stress and learning, with an increase in focus and performance evident in the simulated setting [9].

Much has been written about the clinical environment and its influence on learning [10, 11]. Very little research indicates actual type of learning episodes that contribute to, or hinder, students perceptions of learning. Stress that is associated with positive emotions is called eustress and may result in being attentive to a task, exhilarated, and being fully present [12]. Eustress differs from distress in that it energizes and motivates, may feel exciting and increases focus and performance [13]. Eustress has also been found to have a positive effect on enjoying the workplace [14, 15].

The term stress was deliberately avoided in this study. Previous piloting found that students found the term ambiguous, a finding also supported in the literature [16], where a frequent interpretation of stress is taken to mean distress. In this study we have adopted a more neutral framing by referring to challenge – which, in turn could be positive (eustress) or negative (distress). The definition of challenge used in this study is ‘*Something that needs skills, energy and or determination to deal with or achieve’* [17]. This definition deliberately did not attach any affective qualifier, negative or positive.

It has been proposed that negative and positive emotions may co-occur but positive emotions may have important functions in moderating the stress process and are distinct from those emotions that regulate distress. Positive emotion can help with cognitive tasks such as hypothesis testing [18] and identity formation, potentially strengthening the desire to be a doctor [19]. Holding positive feelings may broaden an individual’s attention and thinking [20]. It is thus not clear in which circumstances challenge, or stress, may benefit learning nor how affect might moderate such influences.

### Conceptual orientation

There are a number of theories that can help us understand these observations, particularly in explaining the role of challenge, and when challenge might be positive for learning. Firstly, the concept of stress related growth and thriving has been evident for over twenty years [21]. Stress related growth espouses that experiencing challenging situations may result in benefits [22]. Vygotsky’s theory of cognitive development [23], proposes that there is a space or distance between actual and potential development of a learner [24]. Disequilibrium presented by challenging tasks are therefore likely to promote learning. Similarly, Mezirow’s theory of transformative learning proposes disorientating dilemmas as starting points for reframing the learning process [25]. Both Mezirow and Vygotsky propose that dissonance is required to promote learning. The difference between what is known and what needs to be known may be perceived as a potential stressor that challenges students.

Secondly the ‘Broaden and Build’ model proposes that positive emotion encourages thinking and exploratory thoughts [26]. Alternatively negative emotions are associated with a narrow survival-oriented response, sometimes referred to as a ‘flight and fight’ response. The ‘Broaden and Build’ model is focused on general enhancement of health rather than its applicability to learning but a similar pathway could occur with learning. Of relevance is that the ‘Broaden and Build’ model relies on the development of active relationships, similar to Vygotsky’s theory of the zone of proximal development (ZPD). In both cases, stretching a learner requires the help of others. The exploratory thoughts, or the build component, in the ‘Broaden and Build’ model also resonates with the concept of growth mind-set and the belief that through effort there is the capacity to grow and develop [27].

Including positive emotions in studies of stress may provide a counterbalance to the predominance of studies that have focused on negative emotions [28, 29]. This study aimed to explore the association of perceived learning with how *one feels* and the degree of challenge. The focus was on perceived learning accommodating the often opportunistic and serendipitous nature of learning in clinical settings. In addition, the circumstances in which learning episodes are both challenging and positive were explored.

## Method

### Context

In a New Zealand medical school, students move from the first 3 years of the medical course that is mainly in the protected classroom and simulated clinical environments, into years 4–6 that are situated mainly in work-place clinical environments based in three campuses. This study was undertaken with a year 4 cohort of students, with a total of 78 students in the year at a single campus.

### Methodology and study design

We asked course organisers, who plan and manage three clinical placements in year 4, to identify 5–10 discrete learning opportunities that they felt might challenge students. These are called course organiser identified learning episodes (COILEs). There was no moderation of the COILEs and they were accepted on face value.

At the end of the first and last courses in year 4, students were invited to a session and were asked, using a paper based survey, to rate the related course COILEs. We used the following criteria and rating scales: 1. the degree of perceived learning, could be rated from no value, very low, low, unsure, high to very high value; 2. the degree of challenge, could be rated from no challenge, very little, little, somewhat, high, to very high; and 3. how they felt about the learning episode (LE) could be rated from very negative, negative, ambivalent, positive, to very positive.

We also asked the year 4 students, to describe in free text a memorable challenging learning episode. These are called self-identified learning episodes (SILEs). The students then rated the SILEs using the same criteria and rating scales as COILEs.

The rating of both COILEs and SILEs was to capture both the intended curriculum opportunities (which course organisers think might be challenging) and the experienced curriculum (what was actually challenging) and to explore any consistencies in associations. Only the student generating the SILE, rated the SILE.

### The nature of associations

We explored if there were significant differences between ratings of SILEs and COILEs using the ranking non-parametric Mann Whitney U test and the associations among the ratings of perceived learning, challenge and affect, for both COILEs and SILEs, using Kendall’s tau-b test.

### The types of learning associated with challenge and learning

We used a mix of qualitative and quantitative approaches to explore this, drawing on both the descriptions of the LEs and their ratings.

Firstly, to explore what makes a learning episode challenging in clinical education, we used grounded theory in conjunction with an inductive interpretivist approach to analyse the 23 COILEs [30, 31]. A grounded theory approach was well-suited as there are no previous studies or explanatory frameworks exploring this area. This analysis was conducted by authors (JR and TW) and firstly involved categorising/theming any COILEs that seem to be similar in nature. The SILE free text data were then read and re-read, and then allocated to one of the established themes or allocated to emergent thematic categories. Where possible SILE quotes were used to give illustrative meaning to the themes generated.

Secondly, we then explored the associations of these categorised LEs with the ratings on perceived learning, challenge and affect. The Kruskal-Wallis Tests, adjusted by the Bonferroni correction for multiple tests, was used to determine significant differences between COILEs within a theme.

Thirdly, we then compared the categories with the ratings to inform whether they should remain together, or be split into separate categories.

The themed LEs were then analysed regarding the frequency values for perceived learning, challenge and affect. *High value* is shown by the proportion of students who gave a rating of high or very high for perceived learning and challenge and positive or very positive for affect. *Low value* is shown by the proportion of students who gave a rating of *low or less* for the learning and challenge rating, and negative or very negative for the affect rating.

Qualitative and quantitative survey-based data were collected using reports from students. Although the collection of quantitative data traditionally leans towards a more positivist research stance a deliberate interpretivist approach was adopted. It is not assumed that there exists an objective reality and the quantitative data were utilised to facilitate emergent properties [32]. In addition the triangulation of the qualitative data collected through free text comments supports this interpretivist orientation.

IBM SPSS version 26 was used for the analysis.

Informed consent was secured prior to any data collection with ethics obtained from the University of Otago Human Ethics Committee (Health), reference number D14/075.

## Results

### Response rates

From 78 students, 51 SILEs were submitted, each rated by the student who identified it. Students were expected to submit SILEs only if they felt they had a memorable example so it is difficult to calculate what an expected response rate would be.

Twenty three COILEs generated by the course organisers are detailed in Table 1. If all students rated all COILEs on each of three modules, 882 responses might be expected; 422 COILE ratings were received. The response rate therefore was 47.5%.

**Table 1:** Clustering and theming COILEs and SILE with illustrative quotes and frequency values

Immersion in **authentic clinical environment**				
*COILEs* • Acute Medicine on-call • Clinical learning outside of 9–5 • Engaging the acute (sick) patient • Range of patients • Role of uncertainty in medicine • Translating ELM clinical examination to the real thing and presenting *SILE illustrative quote* “In medicine working in ED with a helpful / enthusiastic registrar being involved, learning from patients / management of patients.”		Learning	Challenge	Affect
	N	109	108	102
	*Proportion of student ratings*			
	High or very high	74%	64%	62%
	Low /negative	7%	5%	11%
**Simulated** clinical experience				
*COILE* • history taking simulated sessions • simulated GP clinic *SILE illustrative quote* “. ..., assessing acting patient, given responsibility in a situation which we may be in for real in the future.”		Learning	Challenge	Affect
	N	42	42	52
	*Proportion of student ratings*			
	High or very high	92%	55%	95%
	Low /negative	2%	5%	0%
Specific authentic **clinical skills**				
*COILE* • A skill such as IV line placement *SILE illustrative quote* “Taking blood off a patient (rather than a simulation model or a classmate) for the first time.” “Generally when we were asked to function of a member of the team and given tasks to do. Often by the registrar and sometimes in pairs.”		Learning	Challenge	Affect
	N	52	52	52
	*Proportion of student ratings*			
	High or very high	87%	64%	87%
	Low /negative	0%	0%	0%
Specific learning engagement – ***presenting***				
*COILE* • Presentation of a case on ward round • Knowing the basic exam - to do, and to present *SILE illustrative quote* “Presenting patient histories and examinations findings in a professional setting, with time constraints. “		Learning	Challenge	Affect
	N	21	21	21
	*Proportion of student ratings*			
	High or very high	70%	71%	42%
	Low /negative	5%	5%	21%
Specific learning engagement– ***questioning***				
*COILE* • Questioning by staff *SILE illustrative quote* “When asked questions by doctors (mainly positive)” “The time when being questioned by the consultants.” “Being quizzed by kind staff. Sometimes individually and sometimes in a group.”		Learning	Challenge	Affect
	N	18	18	18
	*Proportion of student ratings*			
	High or very high	89%	95%	61%
	Low /negative	0%	0%	11%
**Administrative**/organisational function				
*COILE* • Learning when modules clash • Making choices in a busy time table *SILE* “Getting told off for not being prepared or for not attending a tutorial when we often had clashing commitments. “		Learning	Challenge	Affect
	N	45	46	45
	*Proportion of student ratings*			
	High or very high	13%	33%	9%
	Low /negative	40%	26%	27%
**Balancing learning and patient needs**				
COILE • Balancing patient needs with learning needs *SILE illustrative quote* “By the time we left the room he was very short of breath and tired out from the examination. The experience was challenging because I know that I will need to examine unwell patients as a doctor/medical student, and even though the examination may be inconvenient or uncomfortable patient it will be a necessary part of their healthcare. But (especially as it was for teaching purposes and not for the patient’s benefit) I felt a little bit unhappy with the situation at the time.”		Learning	Challenge	Affect
	N	20	20	20
	*Proportion of student ratings*			
	High or very high	25%	40%	25%
	Low /negative	20%	20%	10%
**Non-on-the job** learning activity				
*COILE* • Reflective essay / assignments *SILE illustrative quote* “:... many of the assignments were very time consuming but not particularly useful, which meant there was less time to independently learn things specific to the areas that we were working in.” COILE Tutorial based sessions - without patients • Pharmacology • Dermatology • Small group work *SILE illustrative quote* “A group tutorial about Parkinson’s syndrome. Team tutorial with students (×3) and consultant.”	Reflective essays/assignment			
		Learning	Challenge	Affect
	N	19	19	19
	*Proportion of student ratings*			
	High or very high	10%	5%	11%
	Low /negative	74%	47%	26%
	tutorial based sessions			
		Learning	Challenge	Affect
	N	51	55	53
	*Proportion of student ratings*			
	High or very high	63%	53%	53%
	Low /negative	6%	4%	4%
**Adapting** to the work place **learning environment**				
*COILE* • Parity (variation) of student experience • Observing what goes on around you and your patient • Making choices about where to position yourself in the clinical environment *SILE illustrative quote* “The transition from rote-learning to the clinical problem solving was challenging.”		Learning	Challenge	Affect
	**N**	85	85	85
	*Proportion of student ratings*			
	High or very high	51%	51%	37%
	Low /negative	19%	17%	13%
**Dealing with emotional components (including death)**				
*COILE* • Unexpected or expected death of a patient *SILE illustrative quote* “Brought together with host GP on an acute call for an unexpected death of a patient (GP run), good exposure and learning by just observing, learning crucial skills involved in dealing with family members.” “The emotional component of the run and all the problems patients have in their lives.”		Learning	Challenge	Affect
	N	13	12	13
	*Proportion of student ratings*			
	High or very high	69%	58%	39%
	Low /negative	8%	8%	39%

### Student ratings of the SILEs and COILEs

LEs identified by students (SILEs) were rated by students significantly higher than those LEs identified by staff (COILEs) (*n* = 482, *p* = 0.001) for perceived learning with over 86% of students rating the SILEs very high or high for perceived learning in comparison with only 56% of COILEs. In addition the rating of affect was significantly different with 48 and 84% of students rating COILEs and SILEs respectively as being positive or very positive (*n* = 477, *p* < 0.001). For challenge, no differences existed with 56% of students rating COILEs and 49% of SILEs, as high or very high challenge (*n* = 484, *p* = 0.336). High perceptions of learning and positive affect seemed to characterise self-identified memorable learning episodes.

### Correlations among perceived learning, challenge and affect

For COILEs there were significant correlations between challenge and perceived learning (tau-b = 0.276, *p* < 0.001, *n* = 429) and between affect and learning tau-b = 0.670, p < 0.001, *n* = 423). For SILEs a significant correlations between challenge and perceived learning (tau-b = 0.356, p 0.007, *n* = 48) and between affect and learning tau-b = 0.329, *p* < 0.012, *n* = 48) was also evident. There was no significant relation between affect and challenge for either COILES (tau-b = 0.113, *p* = 0.07, n = 423) or SILEs (tau-b = 0.072, *p* = 0.582, n = 48).

### Qualitative categorisation of learning episodes

All LEs were placed into 10 categories: Immersion in authentic clinical environments; Simulated clinical experience; Specific authentic clinical skills; Specific learning engagement– *presenting*; Specific learning engagement– *questioning;* Administrative/organisational function; balancing learning and patient needs; Non-on-the job learning activity; Adapting to the work place learning environment; Dealing with emotional components (including death). The categorisation was determined by comparison and discussion between two of the authors (JR and TW) and confirmed by the remaining authors (MT and CJ).

The theme of non-on-the-job learning activities initially included tutorials and a reflective assignment. In looking at the mean ratings there was a significant difference between the reflective assignment and tutorials for learning, challenge and affect (all at p level < 0.001) suggesting that there were two distinct types of learning activity within this theme: course assignment and tutorial teaching. Splitting this theme into two components, reduced the heterogeneity of ratings within this initial theme. The course assignments were considered as being of lower learning value, lesser challenge and affect than the tutorial teaching.

Some LEs could be placed within more than one theme e.g. SILE: “*Being asked by the team to take a history and report the findings. I know they had already seen the patient and knew them but it was still nerve racking and exciting and made me feel like a doctor.*” but a judgement was made to place them in the most salient theme.

Table 1 shows the COILEs and illustrative SILE quotes within each theme, alongside the ratings on perceived learning, challenge and affect.

High perceived learning with positive affect and high challenge is characterized by *active* learning episodes in the real or simulated setting and related to authentic tasks of the doctor e.g. *Immersion in authentic clinical environment, Specific authentic clinical skills.* There are no examples of high perceived learning where there is low challenge or negative affect. Notably, presenting was rated as both high for perceived learning and challenge but the majority of students did not rate affect as being positive.

*Administrative/organisational functions* were associated with a perception of low learning and were of a low challenge and also negative affect. In addition, assignments were felt to have low value for learning and to be of low challenge, particularly the reflective assignment.

The majority of students rated *Dealing with the emotional component of practice* as high for perceived learning and challenge but in respect to affect there was a noticeable split between those who found it a positive or negative experience.

## Discussion

**Fig. 1:**
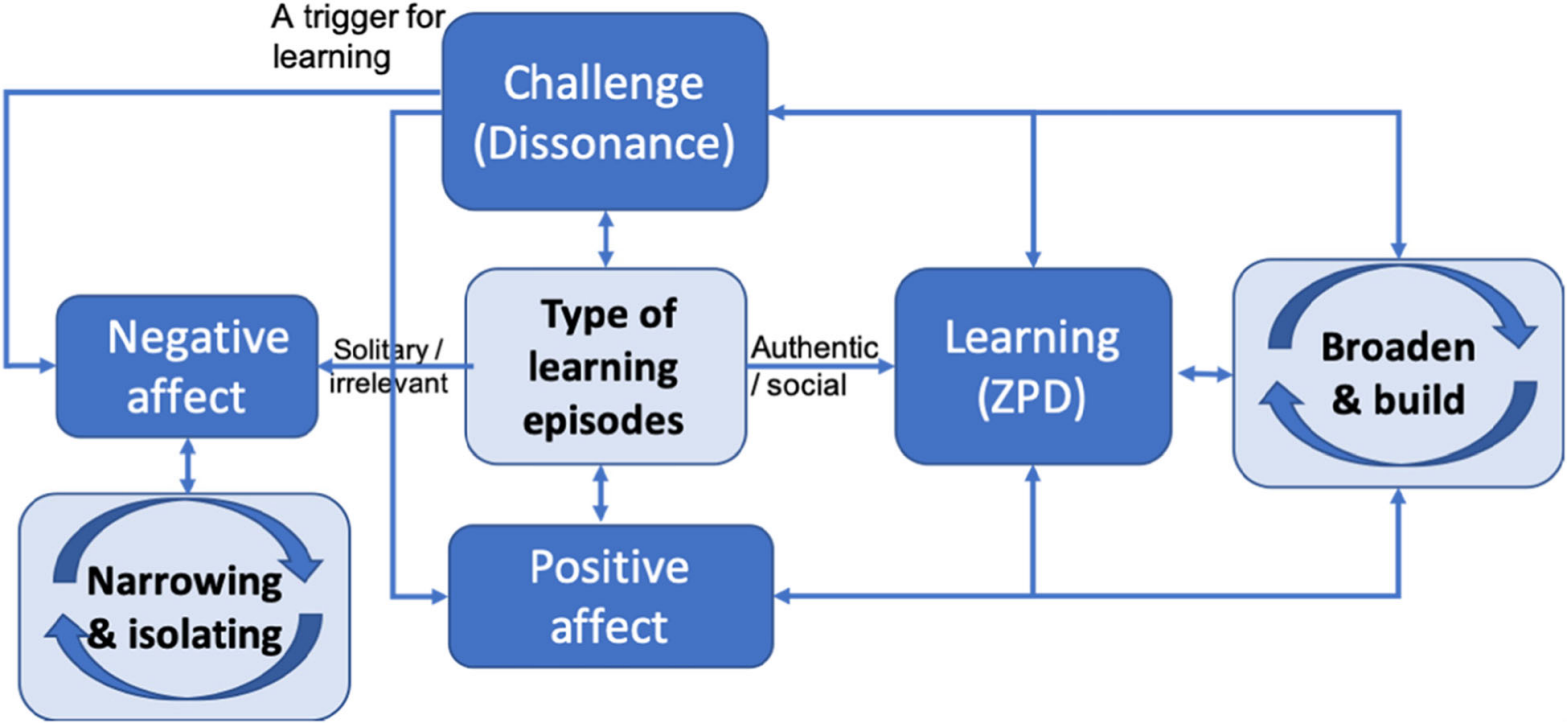
Model of challenge, affect and learning

We have found that both affect and challenge have a beneficial and synergistic impact on perceived learning. There were two key features associated with the types of learning episodes that were most associated with perceived learning (and therefore also seen as both challenging and associated with a positive affect). These were, firstly, that they were seen as occurring within a situation where interactions with others were prevalent and, secondly, that they were representative of authentic clinical activities and or practices of a doctor. A simple model is proposed to encapsulate and explain the relationships found, see Fig. 1:

Learning occurs best when it is within the ZPD [33] and underpinned by a ‘Broaden and Build’ model whereby positive emotion encourages thinking and exploratory thoughts [26]. There can be many triggers for learning but those that create some challenge or dissonance seem the most effective [34]. Learning activities that are solitary or are seen as irrelevant to future practice are regarded negatively and are seen as isolating. The ‘Broaden and Build’ model resonates with work on a growth mind set where how a student perceives a challenge can be positively influential [27].

### Type of learning episodes

It is reassuring that positive associations between perceived learning and affect and between perceived learning and challenge were found irrespective of whether the student self-identified the learning or the learning was identified by a course organiser. This potentially strengthens the generalisability of the positive relationships seen.

Students identified that the type of LE was important for perceived learning. One of the biggest differences between LEs regarded as being valuable for learning, is whether learning included others or was a solitary activity. Immersion in a workplace environment, simulated sessions, practical tasks, questioning and presenting all represent social learning opportunities and or activities, and were of judged to have the greatest learning value. These findings are consistent with social learning theory [35] where learning occurs through observing others and or direct experience. This helps to explain why the relationship with the teacher or clinician is seen as important [36].

In contrast, the least valued activities, such as administrative decisions or writing an assignment, represent solitary activities or activities not obviously aligned to the authentic role of a doctor. Although junior students may initially have little to practically offer a clinical health care team, the focus of providing legitimate authentic roles [37] is imperative, whilst also maintaining patient safety. Much of clinical learning is intimately intertwined with completing a *job* or *task*, associated with work-based learning [38]. In medical education many meaningful experiences are experiential and as such learning occurs on the job and often informally [39]. This helps to explain why authenticity was seen as important.

### Learning

Students indicated a perception that they learnt during challenging LEs. We have interpreted this through Vygotsky’s theory of ZPD. Although ZPD focuses on development we have loosely included development as ‘learning to become a doctor’. We combine the dissonance of challenge and the exposure and support of others to operationalise the ZPD resulting in learning. Vygotsky’s theory relies heavily on the importance of *imitation*. Imitation is not the mindless copying of actions but more deliberate and occurs when development possibilities coincide with available practices to imitate [40]. For example, when a student presents to a senior member of staff they are imitating what they have seen in practice. In addition the zone is attained with relational support, stretching the student further than they could attain on their own. The ability to concisely describe a patient conditions and management is an important developmental clinical art. ZPD is an apt model to adopt given that it has been described for activity learning situations developing mastery of a practice [41]. Better social relationships feed into the concept of ZPD and as such a positive cycle of growth may be evident as seen in the ‘Broaden and Build’ model.

### Challenge

Students perceived greater learning value from LEs that were challenging. We argue that a perceived deficit in knowledge and or skills can frequently be the trigger or stressor for learning itself. This creates potential dissonance necessary for learning growth [24]. Within sport, if a stressor is seen as challenging, as opposed to threatening, individuals are more likely to perform [42]. Clinical students have reported that they believe challenge to be useful for learning [43] and these findings are also consistent with the theory that there has to be disequilibrium to promote learning [25]. Challenging learning episodes offers a useful dissonance.

### Positive affect

The strong association between the perception of learning and positive affect can be interpreted in several ways: the students were positive because they learnt, they were positive due to the learning process, they were positive because they were just in a positive frame of mind. This study supports the findings that students express positive emotions when able to identify as doctors and when given roles equated with being a doctor [44, 45]. Irrespective of the cause or effect, feeling positive may allow a learner to expand the view of themselves and others. Negative affect is to be avoided as it is likely to result in reduced learning, a closing down of learning seeking behaviour [26], and has been shown to be detrimental for practical skill acquisition [19]. Negative emotions are more long lasting, intense and can be more memorable than positive feelings [46]. SILEs were rated higher for perceived learning and positive affect then COILEs. Students identifying LEs invariably chose those that were associated with feeling positive.

The response to dealing with emotional components of practice, including death, had a mixed affective response that was different from other responses. The bimodal distribution between negative and positive may represent a difficultly by students to sperate or identify whether they were responding to the episode itself or the learning associated with the episode. For example as part of a learning episode a student may have felt they learnt how to break bad news, which would evoke a high learning rating, but the actual bad news given may have coloured the affective response.

Challenge and positive affect are both linked to the ‘Broaden and Build’ model [26]. Challenge gives a purpose to expand thinking. Without a trigger, motivation to develop/learn is reduced. Positive affect is likely to enhance learning and encourage an individual’s ongoing attention and thinking, broadening the desire for learning and the development of better social relations and skills [26]. We found that the associated positive or negative affect associated with reflective appraisal by the learner of the LE strongly influenced the perceived learning value of the LE. The ‘Broaden and Build’ model is activated if positive affect is present which then stimulates the seeking of further challenging LEs. This seeking is represented by the build component of the ‘Broaden and Build’ model and also aligns with work on growth-mind sets [27].

Some LEs that were felt to be challenging and that involved others were not always associated with learning or deemed to be positive. Certain opportunities that the students found challenging have been reported in the literature as being associated with negative emotions, for example anxiety when presenting cases [47]. Presenting was reported by a substantial number of students as being a negative experience and as such may represent the variation in individual students, the context of presenting and the staff members receiving the presentation. Even within one discipline there may be different teams that students presented to.

Questioning was deemed to be valuable for learning and generally received positively. However, the questioning style is important and likely to be variable amongst clinical staff and therefore variably interpreted by the student [48]. From a student perspective, managing questioning is not only related to learning but also a student’s desire to project an image to staff [49]. Therefore, rating questioning as positive may have been influenced by the image students felt able to portray. The role of questioning in medicine also has a long tradition and the nuances attached to its value are complex [50].

Although it was useful to explore different types of challenging LEs, it is reassuring that positive associations between perceived learning and affect and between perceived learning and challenge were found irrespective of whether the student self-identified the learning or the learning was identified by a course organiser. This potentially strengthens the generalisability of the positive relationships seen. The finding that SILEs were rated as being of perceived greater value for learning and affect compared with the COILEs indicates that affect may be the most memorable attribute associated with learning. It is unsurprising that learning was perceived as being higher in SILEs as students were requested to identify a memorable learning episode whereas staff identified challenging learning episodes and in hindsight some of these where neither challenging or associated with high learning.

### Limitations

A limitation of the study is the use of self-report. This may be especially pertinent in respect to perceived learning. It has been found in the non-clinical setting, when using a variety of different learning modalities, that students can be inaccurate in the amount of learning perceived as compared to actual learning [51]. It is unclear in the clinical experiential setting whether perceived learning may be accurate, under or over inflated. Samples were relatively small and were recruited from one university. It may not represent a culturally or ability diverse group. In this study students were asked to self-report after an event (the course or a specific learning episode). The outcome of the learning encounter, the learning or performance achieved, may have influenced the reporting of challenge. Where the learner perceived little learning or the challenge may have been reported as being low or high, the challenge never-the-less may have been perceived differently at the onset. In addition, the COILEs selected by course organisers were limited by the organiser’s interpretation of the challenges faced by students. Some course organisers identified administrative challenges that they themselves perhaps had issues, e.g. clashing of activities, that were not perceived by students as challenging and or associated with learning.

This study features that how a student feels (positive or negative affect) has an important influential factor in the perceived learning process. It may be difficult to tease out whether affect influenced perception of the learning or if the learning experience influenced the affect. For example, if a student reported feeling positive on performing an intramuscular injection; did positivity associated with undertaking this learning task result in the perception of learning or did the perception having learnt result in the person feeling positive. This area requires more exploration.

## Conclusion

Challenge and affect are beneficial for learning but are two separate constructs. Negative affect is destructive and should be avoided for learning. Challenge should not be avoided but encouraged. Challenging learning episodes that are the most valued are those that include authentic tasks with social interactions. How positive a learner feels is perhaps the most important facet of learning but not at the expense of feeling challenged.

## Data Availability

The qualitative datasets generated and/or analysed during the current study are not publicly available as the free text response are identifiable and are concerned with the potentially contentious issue of challenge (stress). Some of the data may be misconstrued without contextual awareness. In addition a process of redaction would be required for data that was collected and not directly quoted. However, the data is available from the corresponding author on reasonable request. In addition, checking on consent it was indicated that the data would be securely held and raw data in its entirety only made available to the research team. However, I have no issue in looking into how this sentiment can be maintained whilst still maintaining the clause of informed consent and confidentiality with our data set.

## References

[1] Selye H (1976). 40 years of stress research - principal remaining problems and misconceptions. Can Med Assoc J.

[2] Rudland JR, Wilkinson TJ (2018). When I say … stress. Med Educ.

[3] Le Fevre M, Matheny J, Kolt G (2003). Eustress, distress, and interpretation in occupational stress. J Manag Psychol.

[4] Squiers JJ, Lobdell KW, Fann JI, DiMaio JM (2017). Physician burnout: are we treating the symptoms instead of the disease?. Ann Thorac Surg.

[5] Selye H (1974). Stress without distress.

[6] Cahill L, Gorski L, Le K (2003). Enhanced human memory consolidation with post-learning stress: interaction with the degree of arousal at encoding. Learn Mem.

[7] Maheu FS, Joober R, Lupien SJ (2005). Declarative memory after stress in humans: differential involvement of the beta-adrenergic and corticosteroid systems. J Clin Endocrinol Metab.

[8] Milner B, Squire LR, Kandel ER (1998). Cognitive neuroscience and the study of memory. Neuron.

[9] Girzadas DV, Delis S, Bose S, Hall J, Rzechula K, Kulstad EB (2009). Measures of stress and learning seem to be equally affected among all roles in a simulation scenario. Simul Healthc.

[10] Gallagher P, Carr L, Weng SH, Fudakowski Z (2012). Simple truths from medical students: perspectives on the quality of clinical learning environments. Medical Teacher.

[11] Govender I, De Villiers M. Optimising the learning environment for undergraduate students in the Department of Family Medicine at Sefako Makgatho health Sciences University. S Afr Fam Pract. 2019;136-43.

[12] Simmons BL, Nelson DL (2001). Eustress at work: the relationship between hope and health in hospital nurses. Health Care Manag Rev.

[13] Mills H, Reiss N, Dombeck M. Types of stressors (eustress vs. distress). https://www.mentalhelp.net/articles/types-of-stressors-eustress-vs-distress/. 2008. Downloaded Jan 2019.

[14] Mesurado B, Richaud MC, Mateo NJ (2016). Engagement, flow, self-efficacy, and eustress of university students: a cross-National Comparison between the Philippines and Argentina. J Psychol.

[15] Hargrove MB, Nelson DL, Cooper CL (2013). Generating eustress by challenging employees: helping people savor their work. Organ Dyn.

[16] O'Sullivan G. The Relationship Between Hope, Eustress, Self-efficacy, and Life Satisfaction Among Undergraduates. Soc Indic Res. 2011;101(1):155–72.

[17] "Challenge" *macmillan dictionary*. https://www.macmillandictionary.com/dictionary/british/challenge_1. Downloaded Jan 2019.

[18] Nadler RT, Rabi R, Minda JP (2010). Better mood and better performance: learning rule-described categories is enhanced by positive mood. Psychol Sci.

[19] Brand S, Reimer T, Opwis K (2007). How do we learn in a negative mood? Effects of a negative mood on transfer and learning. Learn Instr.

[20] Fredrickson BL, Cohn MA, Coffey KA, Pek J, Finkel SM (2008). Open hearts build lives: positive emotions, induced through loving-kindness meditation, build consequential personal resources. J Pers Soc Psychol.

[21] Park CL (1998). Stress-related growth and thriving through coping: the roles of personality and cognitive processes. J Soc Issues.

[22] Solcova I, Tavel P. Stress-Related Growth in Two Challenging Conditions. J Hum Perform Extreme Environ. 2017;13(1). https://docs.lib.purdue.edu/jhpee/vol13/iss1/4/. Downloaded July 2019.

[23] Vygotsky L (1978). Mind in society.

[24] Che SM, Spearman M, Manizade A, Lewin R (2009). Chapter 7 Constructive Disequilibrium: Cognitive and Emotional Development through Dissonant Experiences in Less Familiar Destinations. The Handbook of Practice and Research in Study Abroad.

[25] Mezirow J (2002). Transformative learning: theory to practice. New Directions for adult and continuing education.

[26] Fredrickson BL (2004). The broaden-and-build theory of positive emotions. Philosophical Transactions of the Royal Society B: Biological Sciences.

[27] Dweck CS, Yeager DS (2019). Mindsets: a view from two eras. Perspect Psychol Sci.

[28] Folkman S, Moskowitz JT (2000). Stress, positive emotion, and coping. Curr Dir Psychol.

[29] Folkman S (2008). The case for positive emotions in the stress process. Anxiety Stress Coping.

[30] Elo S, Kyngäs H (2008). The qualitative content analysis process. J Adv Nurs.

[31] Glaser B, Strauss A (1999). Discovery of grounded theory.

[32] Babones S (2016). Interpretive quantitative methods for the social sciences. Sociology.

[33] Wass R, Golding C (2014). Sharpening a tool for teaching: the zone of proximal development. Teach High Educ.

[34] Groot F, Jonker G, Rinia M, ten Cate O, Hoff R (2020). Simulation at the frontier of the zone of proximal development: a test in acute Care for Inexperienced Learners. Acad Med.

[35] Bandura A (1977). Social Learning Theory.

[36] Tiberius R, Sinai J, Flak E, NGRea (2002). he Role of Teacher-Learner Relationships in Medical Education. International Handbook of Research in Medical Education Volume 7, edn.

[37] Lave J, Wenger E (1991). Situated learning: legitimate peripheral participation.

[38] Billett S (2006). Relational interdependence between social and individual agency in work and working life. MindCult Act.

[39] Eraut M. Informal learning in the work place. Stud Contin Educ. 2004;26(2):247-73.

[40] Vygotsky L, Hall M, Rieber R (1997). The history of the development of higher mental functions. The collected works of L S Vygotsky: Vol 4.

[41] Chaiklin S, Kozulin A, Gindis B, Ageyev V, Miller S (2003). The zone of proximal development in Vygotsky’s analysis of learning andinstruction. Vygotsky’s educational theory and practice incultural context.

[42] Moore L, Vine S, MRW, Freeman P (2012). The effect of challenge and threat states on performance: an examination of potential mechanisms. Psychophysiology.

[43] Rudland JR, Golding C, Jaye C, Tweed M, Wilkinson TJ (2019). Student belief about the value of challenge. Clinical Teacher.

[44] Dornan T, Pearson E, Carson P, Helmich E, Bundy C (2015). Emotions and identity in the figured world of becoming a doctor. Med Educ.

[45] Kandiah DA (2017). Perception of educational value in clinical rotations by medical students. Adv Med Educ Pract.

[46] Baumeister RF, Bratslavsky E, Finkenauer C, Vohs KD (2001). Bad is stronger than good. Rev Gen Psychol.

[47] Moss F, McManus IC (1992). The anxieties of new clinical students. Med Educ.

[48] McEvoy JW, Shatzer JH, Desai SV, Wright SM (2019). Questioning style and pimping in clinical education: a quantitative score derived from a survey of internal medicine teaching faculty. Teach Learn Med.

[49] Lo L, Regehr G (2017). Medical Students' understanding of directed questioning by their clinical preceptors. Teach Learn Med.

[50] van der Zwet J, de la Croix A, de Jonge L, Stalmeijer RE, Scherpbier A, Teunissen PW (2014). The power of questions: a discourse analysis about doctor-student interaction. Med Educ.

[51] Deslauriers L, McCarty LS, Miller K, Callaghan K, Kestin G (2019). Measuring actual learning versus feeling of learning in response to being actively engaged in the classroom. Proc Natl Acad Sci U S A.

